# Coupling Different Road Traffic Noise Models with a Multilinear Regressive Model: A Measurements-Independent Technique for Urban Road Traffic Noise Prediction

**DOI:** 10.3390/s24072275

**Published:** 2024-04-03

**Authors:** Domenico Rossi, Antonio Pascale, Aurora Mascolo, Claudio Guarnaccia

**Affiliations:** 1 Department of Civil Engineering, Campus of Fisciano, University of Salerno, Via Giovanni Paolo II, 132, 84084 Fisciano, Italy; amascolo@unisa.it; 2 Department of Mechanical Engineering/Centre for Mechanical Technology and Automation (TEMA), Campus Universitário de Santiago, University of Aveiro, 3810-193 Aveiro, Portugal; a.pascale@ua.pt; 3 LASI—Intelligent Systems Associate Laboratory, 4800-058 Guimarães, Portugal

**Keywords:** noise emission models, Road Traffic Noise Models, multilinear regressive approach

## Abstract

Road traffic noise is a severe environmental hazard, to which a growing number of dwellers are exposed in urban areas. The possibility to accurately assess traffic noise levels in a given area is thus, nowadays, quite important and, on many occasions, compelled by law. Such a procedure can be performed by measurements or by applying predictive Road Traffic Noise Models (RTNMs). Although the first approach is generally preferred, on-field measurement cannot always be easily conducted. RTNMs, on the contrary, use input information (amount of passing vehicles, category, speed, among others), usually collected by sensors, to provide an estimation of noise levels in a specific area. Several RTNMs have been implemented by different national institutions, adapting them to the local traffic conditions. However, the employment of RTNMs proves challenging due to both the lack of input data and the inherent complexity of the models (often composed of a Noise Emission Model–NEM and a sound propagation model). Therefore, this work aims to propose a methodology that allows an easy application of RTNMs, despite the availability of measured data for calibration. Four different NEMs were coupled with a sound propagation model, allowing the computation of equivalent continuous sound pressure levels on a dataset (composed of traffic flows, speeds, and source–receiver distance) randomly generated. Then, a Multilinear Regressive technique was applied to obtain manageable formulas for the models’ application. The goodness of the procedure was evaluated on a set of long-term traffic and noise data collected in a French site through several sensors, such as sound level meters, car counters, and speed detectors. Results show that the estimations provided by formulas coming from the Multilinear Regressions are quite close to field measurements (MAE between 1.60 and 2.64 dB(A)), confirming that the resulting models could be employed to forecast noise levels by integrating them into a network of traffic sensors.

## 1. Introduction

When dealing with actual urban area hazards, environmental noise is surely one of the most pervasive and dangerous, with road traffic noise surely being the most prominent of all [1]. As a direct consequence of urbanization increasing, the number of vehicles per inhabitant has constantly grown during the last years, significantly impacting noise pollution in both urban and extra-urban contexts [2], and the big amount of constantly passing vehicles leaves no noise-free spaces. While studies on noise in urban areas were often neglected in the past, they have recently gained remarkable attention from national and international evaluation organizations working to implement strategies for its reduction. For instance, the European Union has outlined a goal to achieve a 30% reduction in the number of people exposed to harmful noise levels by 2030 [3].

It has been undoubtedly provided that exposition to day–evening–night noise levels exceeding 55 dB(A) leads to a series of health issues, listing from the mildest to the most severe: intelligibility during conversations, irascibility, sleep deprivation, mental issues, high blood pressure, and even sudden death [4,5,6,7,8,9,10,11]. Moreover, specific sensible areas are present in urban environments, such as schools. In these places, the control of noise is even more important, since the effects of noise exposure on children can be more severe than on adults [7]. Mitigation actions for the reduction in noise levels in urban areas is a mandatory task, as established by the directive 2002/49/EC [12]. Thus, the accurate assessment of noise levels in a specific area is a fundamental procedure, important for the implementation of targeted action plans. When trying to evaluate noise levels in a given area, two approaches are possible. The most direct–and precise–is to directly measure noise levels with dedicated instrumentation (sound level meters). Nevertheless, on-field measurements are not always the fastest or most economically viable way to proceed. In many conditions, in fact, the morphological arrangement of traffic roads does not permit the installation of fixed stations for noise level monitoring, or sometimes the measurement campaign could be expensive, long, and dangerous. To overcome these issues, the implementation of an effective sensor network in urban areas, that could provide acoustic and traffic data continuously and possibly with low-cost efforts, can be a valid alternative. Such a solution is largely explored in the literature: in [13,14], a system of sensors for the discrimination of traffic noise from anomalous noise events. In [15], a low-cost implementation of urban sensors for urban noise monitoring is described. In [16], a set of wireless acoustic sensors is described for automatic audio event classification. In [17], a general review of wireless sensor systems for smart cities is described. 

When such situations are not implementable, the estimation of noise through a Road Traffic Noise Model (RTNM) is preferable. RTNMs are physical models composed of a Noise Emission Model (NEM) and a sound propagation model [18]. The former assesses the source sound power levels (*L*_W_), while the latter transforms such information into sound pressure levels at receiver points. RTNMs take several parameters as inputs such as the number of vehicles transiting in a certain time period, their categories (light-duty vehicles—LDVs, medium vehicles, heavy-duty vehicles—HDVs), the vehicles’ speeds and/or their accelerations, the distance between the road and the sensible receivers [18]. More complex RTNMs can also take into account other aspects like the presence of roundabouts or intersections (that affect the noise levels due to acceleration maneuvers), the presence or absence of acoustic barriers, and even some climatic aspects like air humidity, temperature, and wind direction [19,20]. It is very interesting to note that the implementation of RTNMs and the use of sensors for data collection are not mutually exclusive. On the contrary, they can be implemented together to obtain the best results. In this idea, sensors provide—in real-time or offline–large quantities of data that are used as input for the predictive models. Some examples of this integration are reported in [21,22,23], and even in [24,25], where large urban area monitoring is exploited.

Different models have been set up by different national institutions, resulting in heterogeneous results when applied in the same context. Among the most used, it is worth mentioning the CoRTN model [26], which is commonly adopted in the United Kingdom, the SonRoad model which has been implemented in Switzerland [27], the NMBP model used in France [28], the ASJ in Japan [29], and the RLS90 in Germany [30], the Harmonoise [31], and Quartieri et al. [32]. Besides all these models, the European Union (EU) has implemented the CNOSSOS one [33,34], which provides a common procedure for the assessment of transportation and industrial noise levels and the consequent development of noise maps, aiming at implementing a stand-alone model for noise assessment in all the European Countries that should receive and use it (by adapting it in some aspects if necessary). Despite the EU’s efforts towards harmonization, the aforementioned national models are still used, especially in academic environments. 

Generally speaking, any implemented noise model suffers from some intrinsic drawbacks, reflecting a variable amount of uncertainty in the final prediction. First of all, any model needs to be calibrated starting from a set of collected data. Such an unavoidable procedure implies that a given RTNM generally performs better in predicting road traffic noise in the same area where its calibration data have been collected. Consequently, when applied in a different scenario (different country, for example), its performance could be severely impaired. Moreover, an RTNM can be generally applied to road traffic conditions similar to the ones of calibration; nonetheless, if the traffic conditions are different from the ones used in the calibration (lower traffic volumes vehicles, lower or higher speeds), the model could perform poorly [18,35]. 

As for the outputs, RTNMs can furnish information in terms of equivalent continuous sound levels (*L*_eq_), percentile levels, or day–evening–night noise levels (*L*_den_). The latter is calculated from the day (*L_day_*), evening (*L_evining_*), and night (*L_night_*) sound pressure levels, as their logarithmic sum which includes a penalty for evening and night hours (the same amount of noise emitted is considered to be more annoying at evening or night than during the day). 

It must be stressed that, although RTNMs represent a valid alternative to long-term noise measurement campaigns, their utilization may be affected by certain factors. Indeed, the equations, constituting the framework of RTNMs, could be difficult to apply, necessitating the development of scripts for their implementation (and the relative programming know-how). In other cases, commercial software is available for the development of noise maps, using a specific RTNM as an algorithm. Therefore, there is a need for procedures that can facilitate the straightforward application of the already-existing RTNMs in the literature, permitting fast usage and reliable results.

For these reasons, the authors implemented a multiregressive technique for traffic noise assessment by calibrating it on computed data instead of real ones. As described in [36,37,38], such a regressive model has the advantage of not needing real data for its calibration. Moreover, the algorithms of generation of its calibration dataset make it potentially applicable to different traffic contexts. On this basis, the authors presented, in this contribution, a new application by coupling the aforementioned multiregressive model with four different existing NEMs (REMEL [39], SonRoad [27], CNOSSOS (and its amendments) [33,34], and NMPB [28]), in turn, coupled with a sound propagation model (namely a simplified formulation of the propagation provided in the CNOSSOS final report [33]). Whereas a comparison between models has already been provided in the literature, a concomitant study on the usage of a multilinear regressive model on different NEMs and sound propagation model, furnishing a modular approach in which a part can be easily substituted by another, is a novelty aspect to the best of our knowledge. The whole code for the generation of the model has been implemented in Python, using the most common packages for data analysis and visualization [36]. It has a low computational cost in terms of memory usage and time of generation (already described in detail in [37]). 

Outputs of the here-presented models are provided as *L*_eq,h_, which is one of the most commonly used noise indicators in the literature, but the proposed methodology has the potential to express the final output as a general function of time, computing the equivalent level at whichever timespan. The validation of the models is provided by applying the equations coming from the multiregression to a set of more than 3000 data elements coming from a Long-Term Monitoring Station (LTMS) by the Université Gustave Eiffel and Unité Mixte de Recherche en Acoustique Environnementale (UMRAE), Nantes [40]. This dataset contains up to seven years of both acoustic and meteorological road traffic data (from 2002 to 2007), collected from a highway located in the city of Saint-Berthevin (France). At the end of the validation process, the *L*_eq,h_ values from the aforementioned dataset were aggregated on an hourly time basis and compared with the estimations provided by the multiregressive linear models application. 

## 2. Materials and Methods

**Figure 1:**
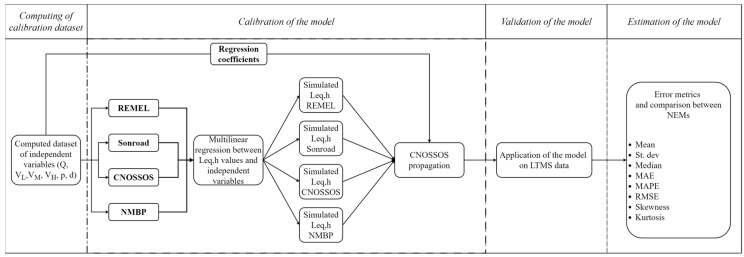
Flowchart of the generation of the model. The computed dataset is used to calibrate the models by computing equivalent hourly noise levels according to the four NEMs investigated, coupled with a simplified version of the sound propagation model provided in CNOSSOS. A multilinear regression technique is then applied, and with the obtained coefficients, the simulated hourly noise levels are computed and compared with the measured ones (from the LTMS dataset) to evaluate the goodness of the proposed approach.

The generation of the model presented in this publication can be divided into four steps: (1) computing of the dataset for the calibration, (2) calibration of the multilinear regression model according to the four considered NEMs coupled with the sound propagation model, (3) validation of the models, (4) estimation of the models, as schematized in Figure 1. Below is the detailed description of each step.

### 2.1. Computing of the Dataset for the Calibration

The dataset used for the calibration of the model is entirely computed, and it is built with sequential steps. The procedure to compute the calibration dataset has been extensively described elsewhere [36,37], and here, a brief recapitulation of the process is furnished. The dataset has been built using Python 3.8, with Jupyter notebook as a compiler. The packages used to develop the code are standard packages for data analysis (*pandas*, *numpy*), for data plotting and visualization (*matplotlib-pyplot* and *seaborn*), and for statistical analysis (*sklearn*). The machine used is a DELL Pc (Intel^®^ Xeon^®^ CPU E3-1245 v5 @3.50 GHz) with 16 GB of RAM installed, 64 bit.

The first step of dataset generation is the building of a series of 200 rows having sequential values of flow, expressed as vehicle per hour (defined as variable *Q*), starting from 10, with incrementing of 10 vehicles at time. From now on, the following steps are intended to be repeated for each row of the dataset. The result of this first step is a column of *Q* spanning from 10 to 2000 vehicles per hour. The second step is the creation of a second column: speed of light vehicles (*V_L_*) filling each row with a randomly extracted value from a minimum of 30 km/h to a maximum of 130 km/h, with a minimum range of 1 km/h. Each value has the same probability of being extracted. The third and fourth steps are the extraction of the speed of medium and heavy vehicles (*V_M_* and *V_H_*). The *V_M_* value is randomly extracted from a minimum of 30 km/h to a maximum value equal to the *V_L_* extracted in the previous step, with a minimum range of 1 km/h. Similarly, the *V_H_* value is randomly extracted from a minimum of 30 km/h to a maximum value equal to the *V_L_* extracted in the previous step, with a minimum range of 1 km. Both *V_M_* and *V_H_* values have the same probability of being extracted between the whole range. The fifth step is the random extraction of a *p*, which represents the percentage over the *Q* of the medium and heavy vehicles, which is composed of a *p_medium_* and a *p_heavy_* value. They are extracted as follows: *p_medium_* value is randomly extracted from a minimum of 0.1% to a maximum of 20.0%%, with a minimum range of 0.1%. All values have the same probability of being extracted. Subsequently, *p_heavy_* is randomly extracted from a minimum of 0.1% to a maximum value equal to 20.0% minus *p_medium_*, with a minimum range of 0.1%. In such a way, the whole *p* value will never exceed 20.0%. The sixth step is the random extraction of *d* representing the source–receiver distance, which spans from a minimum of 10 m to a maximum of 100 m, with a range of 1 m. The last step is the repetition of steps from 2 to 6 for *n* times: in this specific application, *n* is equal to 20. In such a way, a dataset of 4000 rows is built.

### 2.2. Calibration and Validation of the Multilinear Regression Model According to the Four Considered NEMs

The independent variables, generated in the previous step, are used to calculate *L*_eq,h_ values through the four employed NEMs, coupled with a sound propagation model (retrieved from the CNOSSOS model). Particularly, the first step involved the calculation of *L*_W_ for each vehicle category, using the average speed as an input variable. It must be stressed that the REMEL and CNOSSOS models foresee a formulation for the *L*_W_ assessment of medium vehicles. For the other two NEMs, the formulation proposed to assess the *L*_W_ of HDVs was employed also for the medium vehicles. The equations adopted for the *L*_W_ calculation can be retrieved from the model-related reports. Details of such calculations can be found elsewhere [27,28,33,34,39], but for the sake of completeness, the authors report the formulations in Table 1.

Regarding Table 1, it is worth mentioning some important differences between the NEMs used in this work. While the REMEL and SonRoad models compute *L*_W_ through a simple unique formula in which the vehicle speed is the main independent variable, the others are characterized by a more complex structure. Specifically, the CNOSSOS model assesses the propulsion and the rolling (due to the interaction between tires and road pavement) noise contributions separately in each octave band from 63 to 8000 Hz. The contributions of each octave band must be A-weighted and, therefore, logarithmically summed to obtain the overall engine and rolling sound pressure levels. These last ones can be, in turn, logarithmically summed to obtain the overall vehicle sound power level. It is worth reminding that the CNOSSOS model categorizes vehicles into five groups: light-duty vehicles, medium vehicles, heavy-duty vehicles, motorcycles, and the fifth category reserved for alternative vehicles. Since the number of hybrid and electric vehicles is growing in the EU fleet, it will be necessary, then, to update the model including this fifth category. In this regard, Licitra et al. [41] proposed coefficients for electric vehicles in the framework of the CNOSSOS model. Another approach explored in the literature is to use the CNOSSOS formulation for the LDVs by setting the propulsion coefficients to zero, as recently investigated in [42]. Finally, the NMPB model estimates the sound power level from maximum A-weighted sound pressure levels, considering both engine and rolling contributions, during single-vehicle pass-by tests at 7.5 m from the receiver. The rolling noise contribution is distinguished for three road pavement surfaces. In this study, the authors adopted the rolling noise formulation proposed for the third road pavement typology. This choice was driven by the fact that it exhibits characteristics closest to those of the site where the data for the validation process were gathered. It should be noted that correction terms related to acceleration operations, proximity to roundabouts, and intersections, among others, were neglected. The reasons behind this choice are twofold: (i) not all the employed NEMs present such correction terms; (ii) it is difficult to find a robust validation dataset in which acceleration data are available. Nonetheless, other variables as acceleration can be easily included in the proposed approach in future works. It is also noteworthy that CNOSSOS, NMPB, and SonRoad give the possibility to simulate sound power levels for different road surfaces; nevertheless, in this contribution, only the reference surface of each model has been evaluated.

The employed sound propagation method is retrieved and adapted from the CNOSSOS formulations [33]. It must be said that such a model considers the traffic flow as a linear source. At first, the hourly equivalent sound density power levels of the different vehicle categories flows (*L*_WL_, *L*_WM_, and *L*_WH_) are calculated according to the average speeds ($V_{L}$, $V_{M}$, and $V_{H}$),

(1) $${Lw’}_{line,L}=L_{WL}+10\log_{10}\left(\frac{Q_{L}}{1000*V_{L}}\right)$$

(2) $${Lw’}_{line,M}=L_{WM}+10\log_{10}\left(\frac{Q_{M}}{1000*V_{M}}\right)$$

(3) $${Lw’}_{line,H}=L_{WH}+10\log_{10}\left(\frac{Q_{H}}{1000*V_{H}}\right)$$

and then the hourly equivalent sound pressure levels are retrieved by using the linear source propagation formulation:

(4) $$L_{eq,\;L}={Lw’}_{line,L}-10\log_{10}d-8$$

(5) $$L_{eq,\;M}={Lw’}_{line,M}-10\log_{10}d-8$$

(6) $$L_{eq,\;H}={Lw’}_{line,H}-10\log_{10}d-8$$

where *d* is the sound–receiver distance. Therefore, the overall *L*_eq,h_ value comes from the logarithmic sum of the partial contributions:

(7) $$L_{eq,h}=10\log_{10}\left[\left(10^{\frac{L_{eq,L}}{10}}\right)+\left(10^{\frac{L_{eq,M}}{10}}\right)+\left(10^{\frac{L_{eq,H}}{10}}\right)\right]$$

Once the *L*_eq,h_ values are calculated according to the formulas of each NEM and to the propagation, they are used for the multilinear regression. Particularly, an Ordinary Least Squared regression is implemented between the six independent variables (*Q*, $V_{L}$, $V_{M}$, $V_{H}$, *p*, *d*) and the *L*_eq,h_ by using the Python package *sklearn*. The regression formula for each NEM-sound propagation model has the same following structure:

(8) $$L_{eq,h\;simulated}={C_{1}Q+C}_{2}V_{L}+C_{3}V_{M}+C_{4}V_{H}+C_{5}p+C_{6}d+intercept$$

with *C*_1_, *C*_2_, etc., being the coefficients of the multilinear regression model. At this stage, the residuals of the regression are computed and analyzed (the reader can refer to Section 3.2).

The obtained regression formulas are validated by running the model on a field measurements dataset (LTMS) that will be described in the following, and comparing the estimated *L*_eq,h_ with the measured noise levels. Please note that by applying the regression procedure, the authors faced the problem of the uncertainty of the measurement. LTMS data are, in fact, by definition, collected data, and they have an intrinsic uncertainty, which can propagate when a multilinear regression technique is applied to the data. In Section 3.3, a strategy has been implemented to consider such problems, which has also been addressed in the last part of the manuscript (Section 4.3), where the limitations of the study are presented. Moreover, the noise assessment is provided at variable distances, considering the space between the source and receiver as free, without any surrounding building that could be responsible for reflection phenomena.

### 2.3. Estimation of the Performances of the Model

The goodness of the regression models is established by calculating the error as the difference between the measured *L*_eq,h_ and the computed ones, and by studying the errors distributions in terms of statistical metrics such as mean, median, standard deviation, skewness and kurtosis. In addition, the standard metric errors are calculated (Mean Absolute Error–MAE, Mean Absolute Percentage Error–MAPE, and Root-Mean-Square Error–RMSE). All the error metrics and the statistical properties have been computed by using the Python packages *numpy* and *scikitlearn*. Specifically, MAE is defined as follows:

(9) $$MAE=\frac{1}{n}\sum_{i=1}^{n}\left|y_{i}-{\hat{y}}_{i}\right|$$

with n being the number of samples, $y_{i}$ the *i*th measured value, and ${\hat{y}}_{i}$ the *i*th simulated value. MAPE has been computed by Equation (10):

(10) $$MAPE=\frac{1}{n}\sum_{i=1}^{n}\frac{\left|y_{i}-{\hat{y}}_{i}\right|}{max(\varepsilon ,\left|y_{i}\right|)}$$

with ε an arbitrary small yet strictly positive number to avoid undefined results when y is zero. RMSE is computed as follows (Equation (11)):

(11) $$RMSE=\sqrt{\frac{1}{n}\sum_{i=1}^{n}{\left(y_{i}-{\hat{y}}_{i}\right)}^{2}}$$

## 3. Results

### 3.1. Computation and Analysis of the Dataset for the Model Calibration

**Figure 2:**
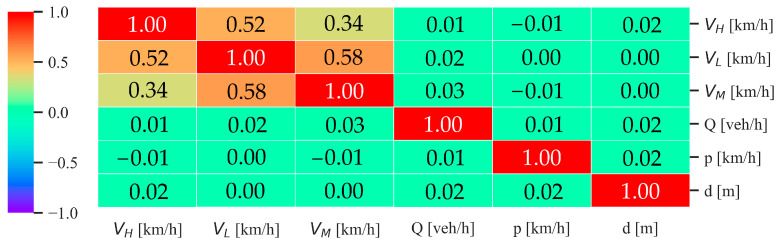
Correlation matrix of the randomly computed dataset for the subsequent model calibration.

The first operation carried out for the generation of the multiregressive model is the computation of the original random dataset. As described in the previous section, this database is computed by joining, in rows, randomly picked values of six independent variables (*Q*, $V_{L}$, $V_{M}$, $V_{H}$, *p*, and *d*). This procedure has the scope of generating a robust and random database to cover a multitude of possible traffic situations. This represents a fundamental step in the model calibration, aiming to avoid potential bias due to lack of information. To augment the possibilities of obtaining a totally random database, a high number of rows is required. Based on observations described in [37], for this application, the authors chose the *n* factor equal to 20, obtaining a final dataset of 4000 entries. Before using it for the calibration of the model, the authors verified that the variables were independently distributed, performing a correlation analysis. The *corr* function of the *pandas* package, on details, correlates each column with all the others by using the Pearson correlation method, obtaining a final correlation value spanning from −1 (maximum inverse correlation) to 1 (maximum correlation), with 0 equal to no correlation; the method of correlation chosen is the standard correlation coefficient. In Figure 2, the correlation matrix is shown, reporting the results of the above-described procedure. 

The correlation matrix shows an obvious maximum correlation of the columns with themselves (central diagonal) and no correlation (green rectangles) when each variable is compared with the others. $V_{L}$, $V_{M}$, and $V_{H}$, have a moderate positive certain degree of correlation, due to the constraints used to generate the dataset. The authors, in fact, imposed that, after certain values, $V_{M}$ and $V_{H}$ cannot be, for every single row, higher than $V_{L}$, to avoid the unlikely situation where all the heavy vehicles, despite the limits fixed by law, run faster than common light vehicles (please refer to Section 2.1 for more details). Hence, apart from the relations between the velocities of the vehicle types, the computed database consists of uncorrelated independent variables. Another important aspect to underline is that the original database just computed corresponds to a *seed* value, which assures its reproducibility. The chosen *seed* value is the same for all the datasets used for the calibration of the model with the four different RTNMs.

### 3.2. Calibration of the Model and Residuals

**Figure 3:**
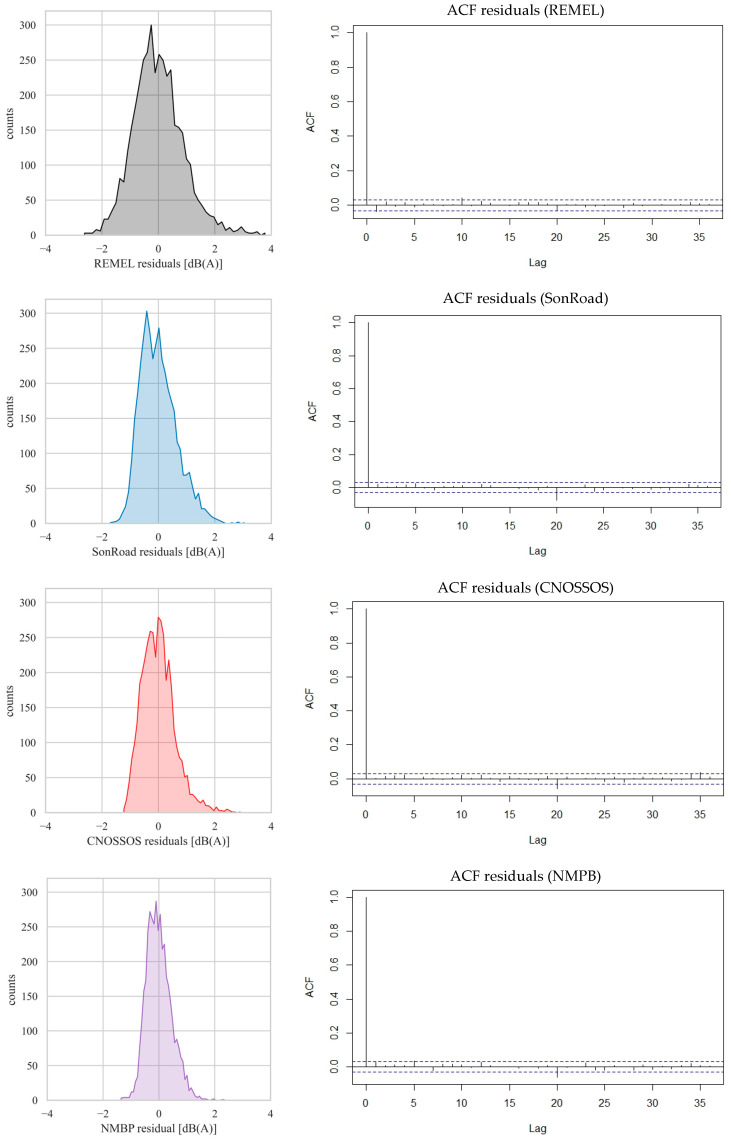
Distributions of the residuals (calibration process) for the different considered models and their autocorrelation plots (dotted lines indicating the level of statistical significance).

As described in Section 2, the four NEMs are coupled with the propagation model. At this stage, the *L*_eq,h_ values are computed using input data from the randomly generated database, consisting of 4000 rows. Thus, the multiregressive model was applied using the information from the database, along with the newly computed *L*_eq,h_ values, resulting in the coefficients reported in Table 2:

Therefore, the residuals of this calibration process were evaluated. They are here defined as the difference between the *L*_eq,h_ values obtained by applying the models (in their basic form) to the database values and the *L*_eq,h_ computed by applying the formulas from the multiregressive technique. The statistical metrics of the residuals coming from the calibration process are shown in Table 3, while their distributions together with the autocorrelation functions are plotted in Figure 3. 

Residuals of calibration are well centered (Figure 3), having a mean value equal to 0.0), with low standard deviation (a minimum of 0.45 dB(A) and a maximum of 0.89 dB(A)). Median values also lie within a narrow interval (from −0.07 to −0.04 dB(A)). Shapiro–Wilk test results indicate that all the residuals are normally distributed (*p*-value ≥ 0.96). The residual distributions are characterized by a positive skewness index, due to a variable amount of data on the right side of the distribution. The kurtosis index is variable, higher for calibrations with REMEL and CNOSSOS but lower with the other RTNMs. Figure 3 reports also the autocorrelograms of the residuals for all the tested models. It is evident that no significant autocorrelation is present as a function of the lag, meaning that no information was left in the residuals and exhibiting a further endorsement of the goodness of the calibration process.

### 3.3. Validation of the Model

The calibration phase is followed by the validation of the models, which involves assessing error metrics using field-measured data.

The dataset used in this paper comes from a Long-Term Monitoring Station (LTMS) installed by the Université Gustave Eiffel (former IFSTTAR) and Unité Mixte de Recherche en Acoustique Environnementale (UMRAE), Nantes [40]. This project was based on the installation of both acoustic and meteorological masts that collected data continuously from 2002 to 2007, in the proximity of a highway in the city of Saint-Berthevin (France). A detailed description of the experimental site is reported in [40], and the data are available upon request. This dataset is originally created from more than 30,000 entries, reporting 15 min *L*_eq,h_ values. For the purposes of this work, hourly *L*_eq,h_ values are needed; therefore, the authors aggregated the data by logarithmically summing all the 15-min entries belonging to the same hour, and excluding the rows with missing values (no missing data imputation method was performed), resulting in a final dataset of 3404 rows complete of all the inputs needed to run the model. Please note that, as described in [36,37], the original LTMS dataset has to be adapted to the model, specifically for the medium and heavy vehicle flows and speeds. 

**Figure 4:**
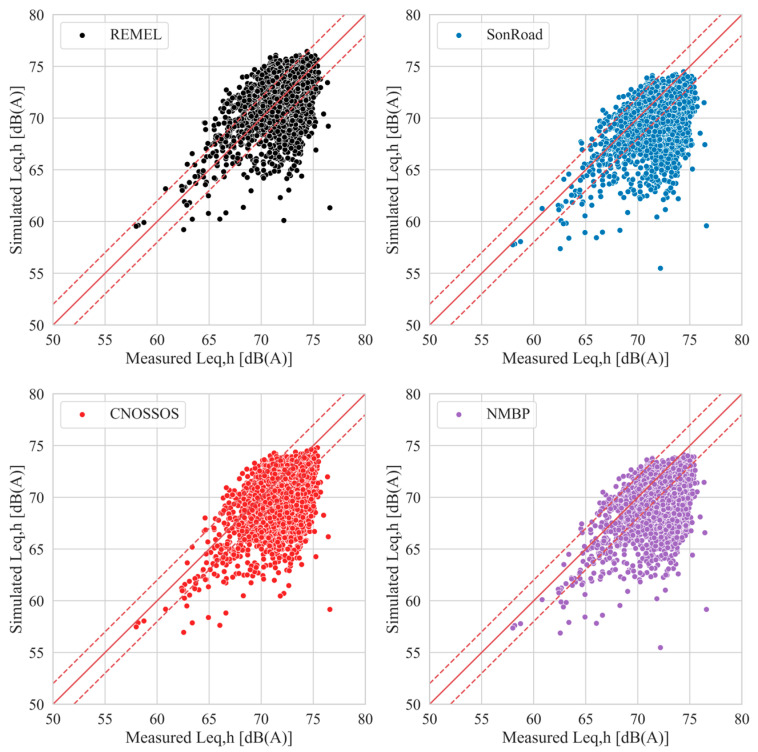
Scatterplots of measured vs. simulated *L*_eq,h_ values of the LTMS dataset for all the four RTNMs after implementation of the multilinear regressive model. The dashed lines represent a ±2 dB(A) interval with respect to the bisector.

Figure 4 reports the measured *L*_eq,h_ values and the simulated ones for each model when the multiregressive linear approach is applied. 

Red lines on the plot show the bisector (continue line) and an interval of ±2 dB(A) (dashed lines). It is visible how the clouds of points all have a similar shape, but their positions vary between the chosen RTNM. Specifically, 71%, 49%, 50%, and 42% of the points are in the region detected by the bisector shifted up and down by 2 dB(A) for REMEL, SonRoad, CNOSSOS, and NMPB, respectively. Such percentages become 84%, 71%, 72%, and 67%, respectively, when the bisector is shifted by 3 dB(A), corresponding to the doubling (halving) of the acoustic pressure. 

**Figure 5:**
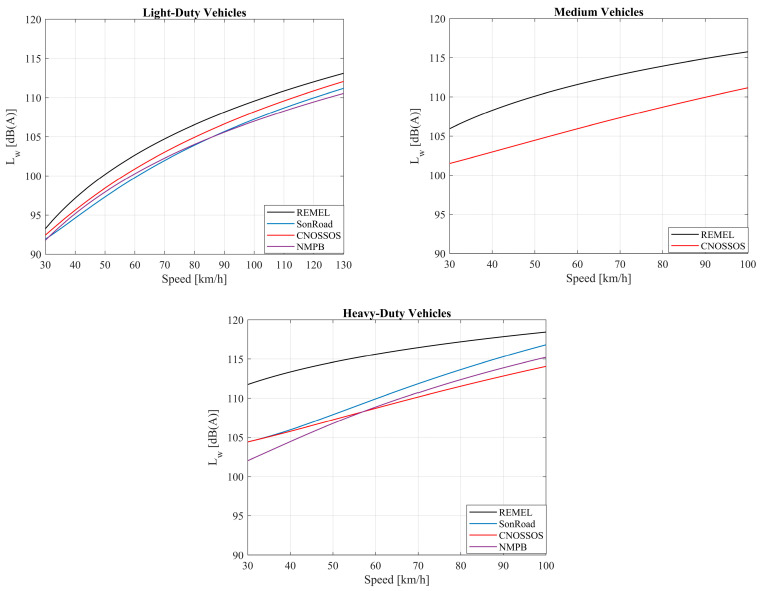
Noise emission curves for LDVs, medium vehicles, and HDVs.

Compared to the REMEL model, the other models tend to underestimate the noise levels. As the sound propagation model is common to all the four employed NEMs, the explanation for such behavior could be attributed to the noise emission curves (expressing the relationship between the vehicle speed and the sound power level) of SonRoad, CNOSSOS, and NMPB, which are lower compared to the ones furnished in REMEL, as it is possible to ascertain from Figure 5.

The metrics related to the distributions of the errors (i.e., the difference between the measured and simulated *L*_Aeq_) are reported in Table 4. REMEL is the model characterized by the lowest mean error, while CNOSSOS, NMPB, and SonRoad present similar performances. The distribution of the errors turns out to be almost symmetric (around the mean), as confirmed by the skewness values close to zero. Moreover, there is a high concentration of errors around the mean, as it is possible to note by the kurtosis values above 1. 

### 3.4. Comparison with RTNMs Application without Regression

After obtaining simulations of *L*_eq,h_ with multilinear regression techniques, a comparison with a straightforward application of RTNMs has been implemented and investigated. As previously stated, one of the issues of the application of the RTNMs is their difficulty of application and the requirement for programming scripts or commercial software for implementations. To overcome these problems, then, a single-time calibration of a multilinear regression technique is helpful in permitting future fast estimations of *L*_eq,h_ values from road traffic data. However, the multiregressive technique must be reliable and present a validation efficiency comparable to that of the RTNMs themselves, so as to make the calibration effort worthwhile. Thus, to estimate the effective validity of the multiregressive approach compared to the sole applications of RTNMs, the authors performed a comparison between the two approaches. The comparison involved statistics of the distributions of simulated *L*_eq,h_ values, error metrics, and computational time investment. This comparison has been carried out on the LTMS dataset.

#### 3.4.1. Statistical Distributions of RTNMs Results

**Figure 6:**
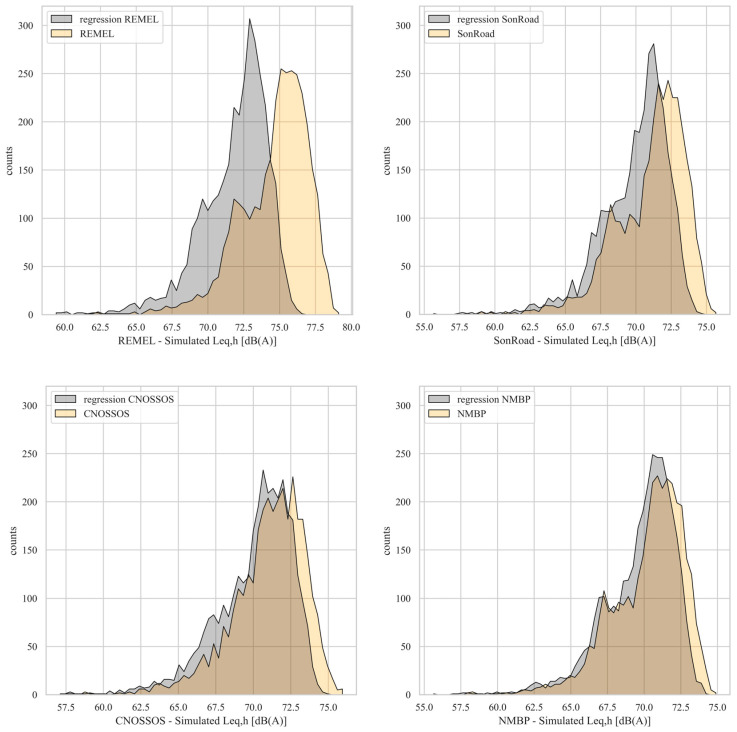
Distribution of simulated *L*_Aeq,h_ from the application of RTNMs with and without the multiregressive approach.

At first, the authors computed the simulated *L*_eq,h_ distributions and the related statistical parameters when the RTNMs were employed with the formulation coming from the multilinear regressive technique and in their original form. Figure 6 overlaps the distributions of the simulated *L*_eq,h_ for the four chosen RTNMs in the two aforesaid approaches, while Table 4 reports the exact values of statistical parameters of the related distributions.

As is evident from the graphs displayed in Figure 6, the simulated *L*_eq,h_ values using the multilinear regressive approach tend to assume slightly lower values compared to the case where RTNMs are applied in their basic form. Consequently, the multiregressive approach may introduce underestimations of the noise levels due to the loss of information introduced by the application of the technique itself. This pattern is further highlighted by the mean values of the simulated *L*_Aeq_, consistently lower when employing the multiregressive linear technique compared to simulations without this approach (the reader can refer to Table 5). In the case of REMEL, the difference between the mean values of simulated *L*_eq,h_ is notably higher than 2 dB(A), highlighting a more pronounced effect. Regarding the shape of the distributions, similarities are observed in both cases, as confirmed by the standard deviation-, skewness-, and kurtosis-related values.

#### 3.4.2. Error Metrics

A comparison between the two approaches was performed also through important error metrics such as MAE, MAPE, MSE, and RMSE (Table 6), computed based on the errors committed for the simulation of the *L*_eq,h_ values on the LTMS dataset. 

As it is possible to note, the MAE values associated with SonRoad, CNOSSOS, and NMPB are slightly lower when the models are applied in their basic form (less than 0.6 dB(A) than in the case in which the multiregression is applied). This is attributed, as mentioned in the previous subsection, to the slight underestimation that the multilinear regressive approach may introduce due to the loss of information during its application. The only exception is REMEL, which appears to experience fewer underestimation issues, at least for the selected case study. Similar trends are observed for RMSE values. In contrast, MAPE values remain consistent across the four considered models.

In general, the performance of the models when the multiregressive approach is applied remains in line with the cases where RTNMs are applied in their basic form, confirming the goodness of the presented methodology.

#### 3.4.3. Computational Efforts Required–CPU Time and Wall Time

**Figure 7:**
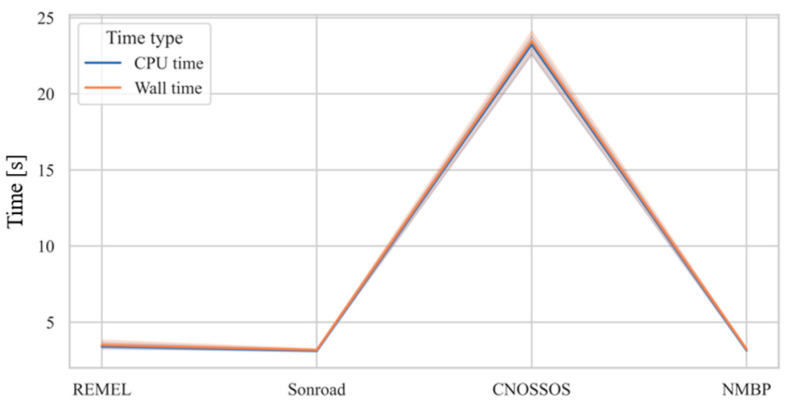
CPU and wall time for implementation of the calibration of the multiregressive approach with the four different RTNMs considered.

The advantage in the implementation of a multiregressive approach can also be found in the computational efforts required to perform the simulation of given *L*_eq,h_ values coming from a set of traffic data. In this subsection, the authors present an evaluation of the time required to compute a fixed number of *L*_eq,h_ with and without multilinear regression implementation for all the four RTNMs investigated. The computer on which the following tests have been performed is the same one described in Section 2, and the tests have been run without any other non-necessary running programs in the background. Two types of time have been evaluated: CPU time (also known as “Execution time”), which is defined as the time needed for the effective execution of the code lines, and wall time, which is the time elapsed from the beginning of the operation to the visualization of the result. These times were evaluated five times for each model (for the estimations of *L*_eq,h_ on the same set of input data), and then the average was computed. It is very important to remember that the implementation of the multiregressive approach is divided into two steps: a calibration step and a validation step. The calibration step, which demands a higher computational effort (increasing with the dimensions of the calibration dataset), only needs to be implemented once. This is because the multiregression coefficients generated can be saved and subsequently used for the validation step. Thus, the authors only compared the validation time of the multiregressive approach with the time needed for the simulation of data by application of each single RTNM. To be complete, indications regarding the calibration time are provided anyway. The time for calibration of the multiregressive model is variable, as shown in Figure 7. 

The implementation of the regressive model, in fact, requires a comparable time with three RTNMs (REMEL, SonRoad, and NMBP), with an average time of 3.27 ± 0.2 s. Calibration time rises with CNOSSOS, which requires 23.39 ± 0.75 s. This can be explained by the fact that the latter is characterized by more complex equations for evaluating the sound power level, resulting in an increased computational burden compared to the other models. The calibration process, then, requires a variable time in the order of seconds. The other part of the process involves using the obtained coefficients to simulate the *L*_eq,h_ values, which are, of course, independent from the RTNMs used for the calibration. 

The simulation of the *L*_Aeq,h_ values starting from the coefficient obtained from the multiregression requires less CPU time than the RTNMs alone which, as remembered in Section 1, can be difficult to implement or require dedicated software. The difference is in the order of milliseconds, which may seem to be irrelevant, but it can become significant when the number of *L*_eq,h_ values to be simulated increases. It also has to be noted that the variation in the time needed for the calculation of *L*_Aeq,h_ is more stable when implementing regression than when applying only RTNMs. This may be due to the higher number of lines to be read from the compiler than the ones of the regression technique.

## 4. Discussion

### 4.1. Dataset for Calibration

The simulation of noise levels coming from road traffic data has dramatic importance when real on-field measurements cannot be implemented. Many models have been implemented over time to best transform the input data to noise levels close to real ones. In this contribution, an approach based on a multiregressive technique has been implemented, to retrieve a model that can result in a reliable output. The calibration of the model has been based on a computed dataset of six independent variables; this approach has a double meaning: (i) it can help in conditions where no real field data can be collected, and (ii) it helps in virtually simulating any type of traffic situation that could occur in a given scenario. Such dataset length can be varied according to the necessity, and it has been established in 4000 entries to assure reliability and repeatability. A smaller dataset, in fact, could help in a reduction in the final total computation time but results in more unstable results since the output coefficients would fluctuate over the repetitions. In the present manuscript, we also demonstrated that the six variables used for the simulation of *L*_eq,h_ are independent between them, which is a mandatory condition for a correct multilinear regression. 

### 4.2. Model Performances

Error metrics used to evaluate the model have shown interesting aspects of the multilinear regression technique when compared to the RTNMs in their basic formulation. At first, the final results are impaired by an error that is similar to or slightly higher than the one of the RTNMs applications. This loss of accuracy in the regression model can be explained by the multilinear regression technique itself, which adjusts all the coefficients to best minimize the error of each linear regression. Due to the number of variables, such fitting inevitably requires relative adjustments that may lead to the loss of information. Apparently, this procedure could also require a very high amount of time to be implemented, and thus ultimately not be convenient over the application of the RTNMs as they are. However, the simulation of the noise levels (in our application hourly levels) is faster once the regression coefficients have been established. This may be a high advantage when simulating a very high number of road traffic data, which is more and more common with the emerging recording techniques. Another aspect to take into consideration is the simplicity of the simulation of noise levels by using the multiregression coefficients compared to the application of RTNMs, which requires a lower computation time. 

### 4.3. Connections with Sensors Networks

The road traffic noise model proposed can be calibrated on a computed dataset to cover multiple traffic conditions, as presented in this paper, or on any field measurements dataset. The latter option, of course, may be affected by the measurement location features. Anyway, in both cases, the model needs to be validated on a large dataset collected by sensors networks, as was done in the paper using the LTMS sensors data. Thus, the outputs of monitoring networks and digital infrastructures are essential for a proper development of the proposed methodology. 

Moreover, the idea of building an IoT framework for assessing noise impact on a given area with this approach can surely be developed. A network of sensors continuously collecting road traffic data related to variables used in the regression could be interfaced with the proposed methodology, to output equivalent noise levels in near real-time, thanks to the very low computational cost. The outputs can then be pivoted to any software able to spatialize the data, such as any Geographic Information System (GIS) framework, to produce noise maps.

### 4.4. Final Evaluation of the Model and Its Limitations

In a comprehensive evaluation of all the aspects of this research, the implementation and usage of the multilinear regression technique is finally advantageous since it is reliable, simple, and based on a solid calibration dataset that does not require real measurement to be built. The calibration dataset used is a key point of the whole procedure since it gives the possibility to build a solid model for road traffic noise simulation without any on-field measurements. This is important in situations where measurements are difficult to be carried out but also when the evaluation of a future noise impact is at stake. Properly simulating the independent road traffic variables could, in fact, aid in forecasting the impact of traffic, facilitating the accurate evaluation of the infrastructure arrangement. The presented model also presents drawbacks, and they are mainly addressed in the loss of information during the generation of the coefficients of the regression. During this operation, in fact, an amount of information is inevitably sacrificed for the sake of simplicity. By observing the error metrics, moreover, it can be seen that the final accuracy of the model is strongly dependent on the NEMs used for the calibration. Up to now, this forces the user to conduct multiple calibrations to find the best NEMs for the fitting (just like the application of more than one RTNM is often required for the best results). A second intrinsic limitation of the study is that the application of a regressive model on collected data has to inevitably face the problem of uncertainty of measure, already mentioned in Section 2.2. Future steps of this work will deepen the statistical analysis and the interval of confidence approach to assure a more coherent comparison with the real data used for model validation. Another aspect to bear in mind is that the validation process, at this stage, has only been pursued on a single database. One of the first next steps of this research, then, will be testing the validity of the model on different traffic conditions, following the incorporation of additional variables such as different road surfaces into the noise emission models, as well as ground and obstacle reflections, atmospheric absorption, among others, into the sound propagation model. A last limitation aspect to take into consideration is that the employed NEMs have all been built in the framework of a combustion engine fleet of vehicles, but recently, the composition of fleet is changing due to a growing number of hybrid and electric cars. Anyway, the modular structure of the proposed approach allows to easily integrate new versions of noise emission models that will consider the different emission curves for electric vehicles as soon as they become available.

## 5. Conclusions

The multilinear regressive approach presented in this study yields robust simulations of *L*_eq,h_ values. A computed dataset was employed to calibrate the models, while the validation process was performed by using robust and reliable traffic and noise data from a large database, available in the literature. A detailed comparison has been presented by using four different RTNMs for the calibration (resulting from the combination of four NEMs and a simplified sound propagation model). A validation on a field measurement dataset, built with the adoption of several sensors, has been performed. The results demonstrated that the proposed approach is suitable for the estimation of noise levels (MAE ranging between 1.60 and 2.64 dB(A)), particularly when compared with the application of the models in their basic form (MAE values between 1.85 and 2.89 dB(A)). While the multilinear regression approach may result in a loss of information, causing a slight underestimation of the noise levels on one side, on the other side, it leads to obtaining easy formulas to be applied after an initial calibration process. This also has repercussions on the computational burden associated with the applications of the models.

Finally, it must be stressed that the proposed methodology could serve as support for a network of traffic sensors (collecting data in terms of traffic volumes and speed), allowing a fast and online estimation of noise levels, without the aid of sound level meters.

## Figures and Tables

**Table 1:** Calculations of *L*_W_ according to the four NEMs used.

REMEL [39]	$L_{WL}=31.130\;\log_{10}\left(V_{L}\right)$ + 12.700 $L_{WM}=18.765\;\log_{10}\left(V_{M}\right)$ + 43.967 $L_{WH}=12.831\;\log_{10}\left(V_{H}\right)$ + 58.270
SonRoad [27]	$L_{WL}=28.5+10\log_{10}\left[10^{\frac{7.3+35\times \log_{10}V_{L}}{10}}+10^{60.5+\log_{10}\left(1+{\left(\frac{V_{L}}{44}\right)}^{3.5}\right)}\right]$ $L_{WM}=28.5+10\log_{10}\left[10^{\frac{16.3+35\times \log_{10}V_{M}}{10}}+10^{74.7+\log_{10}\left(1+{\left(\frac{V_{M}}{56}\right)}^{3.5}\right)}\right]$ $L_{WH}=28.5+10\log_{10}\left[10^{\frac{16.3+35\log_{10}V_{H}}{10}}+10^{60.5+\log_{10}\left(1+{\left(\frac{V_{L}}{56}\right)}^{3.5}\right)}\right]$
CNOSSOS [33,34]	$L_{W,i}=10\log_{10}\left(10^{\frac{L_{W,rolling,i}}{10}}+10^{\frac{L_{W,propulsion,i}}{10}}\right)$ $\mathrm{with}\;L_{W,\;rolling}$$\;\mathrm{and}\;L_{W,\;propulsion}$ given in [33,34] for each vehicle category and each frequency octave band (*i*) from 63 to 80,000 Hz
NMBP [28]	$L_{W}=10\log_{10}\left(10^{\frac{L_{W,rolling}}{10}}+10^{\frac{L_{W,propulsion}}{10}}\right)+20\log_{10}\left(d_{ref}\right)+10\log_{10}\left(2\pi \right)$ $\mathrm{with}\;L_{W,\;rolling}$$,\;L_{W,\;propulsion}$$,\;\mathrm{and}\;d_{ref}$ given in [28] for each vehicle category

**Table 2:** Multiregressive model coefficients.

	REMEL	SonRoad	CNOSSOS	NMBP
***C*_1_**	10.06	10.03	10.03	10.02
***C*_2_**	10.15	12.53	15.65	12.96
***C*_3_**	1.41	3.05	0.52	3.02
***C*_4_**	0.33	0.91	0.12	1.21
***C*_5_**	3.14	3.36	1.28	2.46
***C*_6_**	−12.81	−12.84	−12.85	−12.83
***INT***	31.87	20.51	22.43	19.65

**Table 3:** Statistical metrics of residual distributions (calibration process).

	REMEL	SonRoad	CNOSSOS	NMBP
Mean [dB(A)]	0.00	0.00	0.00	0.00
St dev [dB(A)]	0.89	0.64	0.59	0.45
Median [dB(A)]	−0.06	−0.07	−0.04	−0.05
Mode [dB(A)]	−1.43	−0.37	−0.67	−0.14
Min [dB(A)]	−2.68	−1.76	−1.27	−1.39
Max [dB(A)]	3.84	3.09	2.94	2.37
Shapiro	0.98	0.97	0.97	0.98
Skewness	0.56	0.66	0.82	0.56
Kurtosis	0.96	0.40	1.33	0.67

**Table 4:** Metrics related to the distributions of the errors.

	REMEL	SonRoad	CNOSSOS	NMBP
Mean [dB(A)]	0.15	2.15	2.01	2.40
St dev [dB(A)]	2.15	2.19	2.24	2.18
Median [dB(A)]	−0.02	1.95	1.87	2.24
Min [dB(A)]	−6.15	−4.12	−4.29	−3.85
Max [dB(A)]	15.20	17.00	17.42	17.42
Shapiro	0.97	0.97	0.98	0.97
Skewness	0.67	0.71	0.53	0.67
Kurtosis	1.79	1.96	1.24	1.85

**Table 5:** Statistical properties of measured and simulated *L*_eq,h_ distributions for the RTNMs with and without the multiregressive approach.

	Measured	REMEL		SonRoad		CNOSSOS		NMBP	
		Mult. Regr.	w/o Mult. Regr.	Mult. Regr.	w/o Mult. Regr.	Mult. Regr.	w/o Mult. Regr.	Mult. Regr.	w/o Mult. Regr.
Mean [dB(A)]	72.09	71.93	74.59	69.94	71.03	70.08	70.97	69.68	70.23
Std [dB(A)]	2.00	2.35	2.42	2.38	2.46	2.53	2.48	2.42	2.47
Median [dB(A)]	72.47	72.46	75.10	70.48	71.58	70.62	71.33	70.23	70.74
Shapiro	0.89	0.92	0.93	0.92	0.93	0.93	0.94	0.92	0.93
Skewness	−1.69	−1.25	−1.14	−1.25	−1.11	−1.15	−1.14	−1.23	−1.10
Kurtosis	4.87	2.49	2.05	2.50	1.78	1.83	2.22	2.31	1.77

**Table 6:** Error metrics of simulated *L*_eq,h_ for the RTNMs with and without the multiregressive approach.

	REMEL		SonRoad		CNOSSOS		NMBP	
	Mult. Regr.	w/o Mult. Regr.	Mult. Regr.	w/o Mult. Regr.	Mult. Regr.	w/o Mult. Regr.	Mult. Regr.	w/o Mult. Regr.
MAE [dB(A)]	1.60	2.89	2.44	1.85	2.39	1.88	2.64	2.29
MAPE [%]	0.02	0.04	0.03	0.03	0.03	0.03	0.04	0.03
RMSE [dB(A)]	2.16	3.33	3.07	2.47	3.00	2.41	3.24	2.89

## Data Availability

The data used for the calibration are available upon request from the corresponding authors. The data used for the validation are available upon request at http://ltms2002-2007.ifsttar.fr/ (accessed on 17 September 2023) [31].
